# Impact of Empagliflozin Versus Dapagliflozin on Left Ventricular Remodeling in Heart Failure Patients: A 1‐Year Comparative Study

**DOI:** 10.1002/clc.70192

**Published:** 2025-09-18

**Authors:** Mahmoud Balata, Marc Ulrich Becher, Marwa Hassan, Mohamed Rady, Shady Rashed, Usama Alkomi, Marian Christoph, Karim Ibrahim, Akram Youssef

**Affiliations:** ^1^ Heart center University Hospital Bonn Bonn Germany; ^2^ Department of Internal Medicine and Cardiology University hospital Rostock, Rostock; ^3^ Department of Internal Medicine and Cardiology Städtisches Klinikum Solingen Solingen Germany; ^4^ Department of immunology Theodor Bilharz Research Institute Giza Egypt; ^5^ Department of Internal Medicine and Cardiology, City Chemnitz Teaching Hospital Medical Campus of the Technical University of Dresden Chemnitz Germany

**Keywords:** dapagliflozin, empagliflozin, left ventricular remodeling, SGLT2 inhibitors

## Abstract

**Background:**

Sodium‐glucose cotransporter‐2 inhibitors (SGLT2is) reduce cardiovascular mortality and heart failure (HF)‐related hospitalizations in HF patients. However, the mechanisms underlying these benefits remain unclear, and it is uncertain whether empagliflozin and dapagliflozin have differential effects on cardiac structure and function.

**Aim:**

This study aims to compare the effects of these two SGLT2is on left ventricular echocardiographic parameters in HF patients over 1 year.

**Methods:**

This retrospective study included 558 consecutive HF patients newly prescribed either dapagliflozin or empagliflozin. Key echocardiographic parameters, such as peak E‐wave velocity, E/e' ratio, left atrial volume index (LAVI), LV end‐diastolic and end‐systolic volumes (LV‐EDVI, LV‐ESVI), LV mass index (LV‐MI), relative wall thickness (RWT), LV sphericity index (LV‐SI), and ejection fraction (LVEF), were measured at baseline and after 1 year.

**Results:**

At 1‐year, significant reductions were observed only in the empagliflozin group for peak E‐wave velocity (mean difference = −12.76 cm/s, 95% CI: −16.26 to −9.27, *p* < 0.001), E/e' ratio (mean difference = −3.04, 95% CI: −4.17 to −1.91, *p* < 0.001), and LV sphericity index (LV‐SI; mean difference = −0.01, 95% CI: −0.02 to −0.0005, *p* = 0.040). Both SGLT2is significantly improved E‐wave deceleration time, LAVI, LV‐EDVI, LV‐ESVI, LV‐MI, and LVEF. Neither medication produced significant changes in RWT, and no significant differences were noted between groups regarding HF hospitalizations or all‐cause mortality.

**Conclusion:**

Empagliflozin demonstrated more pronounced effects on LV remodeling markers, including peak E‐wave velocity, E/e' ratio, and LV‐SI, compared to dapagliflozin. These findings suggest potential efficacy differences between SGLT2is, highlighting the need for future randomized comparative studies.

## Summary

- What is known?
  ∘ SGLT2is like empagliflozin and dapagliflozin reduce HF‐related hospitalizations and cardiovascular mortality in patients with reduced and preserved ejection fraction. These benefits appear independent of their glucose‐lowering effects.
- What is new?
  ∘ Empagliflozin demonstrated more significant effects on specific LV remodeling markers, such as peak E‐wave velocity, E/e' ratio, and LV sphericity index, compared to dapagliflozin, suggesting potential differences in efficacy among SGLT2is in HF.
- What is next?
  ∘ Future randomized studies are needed to compare SGLT2is directly, examining the specific mechanisms underlying their effects on LV remodeling to guide personalized HF treatment strategies.

## Graphical abstract

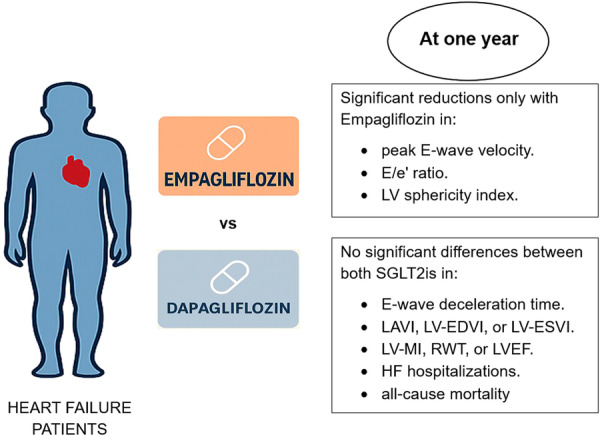

**Abbreviations**
HF: heart failure
HFpEF: heart failure with preserved ejection fraction
HFrEF: heart failure with reduced ejection fraction
LAVI: left atrial volume index
LV: left ventricle/ventricular
LVEDV: left ventricular end‐diastolic volume
LVEF: left ventricular ejection fraction
LVESV: left ventricular end‐systolic volume
LVMI: left ventricular mass index
LV‐SI: left ventricular sphericity index
NT‐proBNP: N‐terminal pro‐B‐type natriuretic peptide
RWT: relative wall thickness
SGLT2i: sodium‐glucose cotransporter‐2 inhibitor

## 1 Introduction

Sodium‐glucose cotransporter‐2 inhibitors (SGLT2is) have recently emerged as a standard of care in heart failure (HF) management [1]. Clinical trials such as EMPEROR‐Reduced and DAPA‐HF have demonstrated that SGLT2is, specifically empagliflozin and dapagliflozin, respectively, significantly reduce the risk of cardiovascular death and HF‐related hospitalizations in patients with chronic HF and reduced left ventricular ejection fraction (HFrEF) [2, 3]. Subsequent trials, including EMPEROR‐Preserved and DELIVER, have further extended these benefits to patients with HF and preserved ejection fraction (HFpEF), showing efficacy across a broader range of HF patients, regardless of diabetic status [4, 5].

However, the precise mechanisms underlying these cardiovascular benefits remain unclear and are likely independent of their glucose‐lowering effects [6, 7]. This is evidenced by the absence of similar HF benefits in other glucose‐lowering medications [6, 7]. Furthermore, dapagliflozin has been shown to improve clinical outcomes in non‐diabetic HF patients without affecting HbA1c levels [8]. These findings suggest mechanisms beyond glycemic control.

Many studies have examined the effects of SGLT2is' on cardiac structure and function to clarify the mechanisms behind their cardiovascular benefits, yet the exact processes remain unclear [8, 9]. Additionally, it is unclear whether empagliflozin and dapagliflozin exert similar effects on cardiac structure and function in HF patients. Medications within the same class can differ in their efficacy; for instance, carvedilol reduces mortality in HF patients by more than 15% compared to metoprolol [10]. Similarly, previous studies suggest that empagliflozin may lead to greater reductions in weight, blood pressure, and cholesterol than dapagliflozin, especially in diabetic patients [11]. This suggests that individual SGLT2is might impact cardiac structure and function differently, a possibility that remains underexplored.

To address these gaps, the present study aims to explore the impact of SGLT2is, specifically dapagliflozin and empagliflozin, on echocardiographic parameters in HF patients to better understand the mechanisms underlying their cardiovascular benefits. Additionally, it compares the effects of both drugs on these parameters over a 1‐year follow‐up period.

## 2 Methodology

### 2.1 Study Population

This study was a retrospective analysis of all consecutive HF patients newly prescribed SGLT2is at the Heart Center Bonn, University Hospital Bonn, Germany, between August 2019 and May 2023. Patients were eligible for inclusion if they were 18 years or older, had a confirmed diagnosis of HF, and were receiving guideline‐directed medical therapy for HF [1]. Additionally, they needed to have complete echocardiographic measurements taken before and 1 year after initiating SGLT2i (empagliflozin at a daily dose of either 10 or 25 mg, or dapagliflozin at a fixed dose of 10 mg daily). These measurements included Peak E‐wave, E/e' ratio, E/A ratio, E‐wave deceleration time, left atrial volume index (LAVI), left ventricular end‐diastolic volume index (LV‐EDVI), end‐systolic volume index (LV‐ESVI), left ventricular mass index (LV‐MI), left ventricular relative wall thickness (RWT), left ventricular sphericity index (LV‐SI), and left ventricular ejection fraction (LV‐EF).

Exclusion criteria included the absence of a HF history, lack of follow‐up visits, prior prescription of or intolerance to SGLT2is, discontinuation or switching of SGLT2i treatment during follow‐up, history of hypoglycemia, type 1 diabetes mellitus, an estimated glomerular filtration rate (eGFR) below 30 mL/min/1.73 m², and the presence of terminal illness. Ethical approval for the Bonn Registry was obtained from the local ethics committee, and the study complied with the Declaration of Helsinki and its amendments.

### 2.2 Echocardiographic Measurements

Echocardiography was performed using either GE Vivid E95, GE Vivid E90, GE Vivid S70N (GE Healthcare, Chicago, Illinois, USA), or Philips EPIQ CVxi (Philips Healthcare, Amsterdam, Netherlands) machines. The LV‐SI was calculated by dividing the mid left ventricular (LV) diameter by the LV long‐axis length at end diastole, both measured in the apical 4‐chamber view. The LV‐MI was calculated at end diastole using the Devereux formula: LV‐MI = 0.8 (1.04 × [interventricular septal thickness + mid LV diameter + posterior wall thickness]³−[mid LV diameter]³) + 0.6 g, divided by the body surface area [12]. The RWT was calculated using the formula RWT = 2 × posterior wall thickness/mid LV diameter at end diastole. Cardiac chamber quantification and evaluation of systolic function were performed according to international guidelines [13].

### 2.3 Statistical Analysis

Continuous variables were expressed as means ± standard deviation or as medians with interquartile ranges, depending on the data distribution. Categorical variables were presented as frequencies and percentages. Group comparisons between baseline and 1 year after SGLT2i initiation were conducted using the Wilcoxon signed‐rank test or paired *t*‐tests, based on the distribution of the data. For time‐to‐event analyses, *p* values derived from a log‐rank test were used for between‐group comparisons. Hazard ratios (HR) and associated 95% confidence intervals (CI) for the treatment effects were estimated with the use of a Cox regression model. Statistical significance was set at a two‐sided *p* value of < 0.05. All analyses were performed using IBM SPSS Statistics, version 26.0 (IBM Corp, Armonk, NY, USA).

## 3 Results

### 3.1 Patient Characteristics

**Figure 1:**
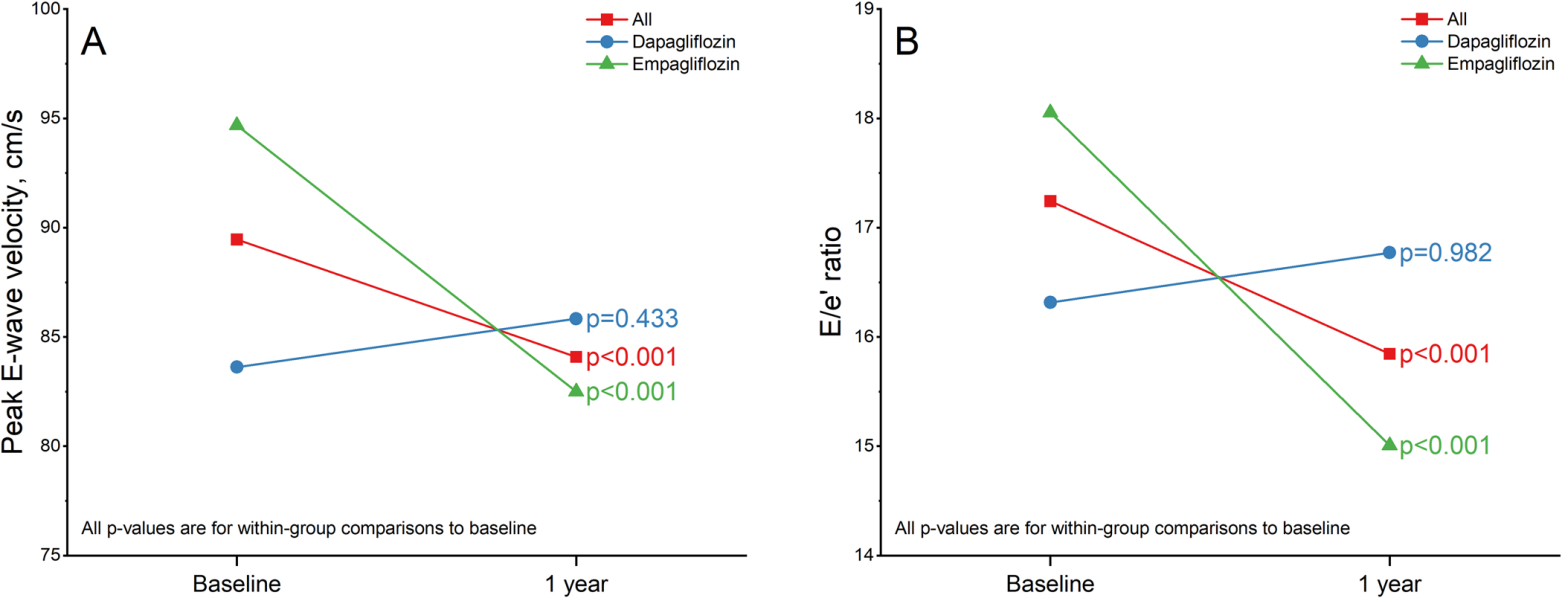
Peak E‐wave velocity (A) and E/e' ratio (B) at baseline and after 1 year. After 1 year, empagliflozin was associated with a significant reduction in peak E‐wave velocity and E/e' ratio (green, *p* < 0.001), whereas no significant changes were observed with dapagliflozin (blue, *p* = 0.433 and *p* = 0.982, respectively).

Among the cohort of 558 patients who received SGLT2i, 294 (53%) were prescribed empagliflozin (Supporting Information S1: Figure 1). The patients had a median age of 62 years (IQR 52−75 years), with 399 (72%) being male. Among these patients, 299 (54%) had coronary artery disease, while 202 (36%) had type 2 diabetes mellitus. The median LV‐EF was 36% (IQR 30%−46%), with 373 (67%) of patients exhibiting an LV‐EF of ≤ 40%. The median NT‐proBNP level was 1256 pg/mL (IQR 613−3512 pg/mL). Table 1 provides a comprehensive summary of the baseline patient characteristics.

**Table 1:** Baseline patient characteristics.

	All	Dapagliflozin	Empagliflozin	*p* value
No. of patients	558	264	294	
Age, year	62 (52−75)	63 (57−76)	61 (45−72)	< 0.001
Male sex	399 (72%)	184 (70%)	215 (73%)	0.398
BMI, kg/m²	28.1 (23.8−32.9)	27.5 (24.2–30.7)	29.3 (23.8−34.3)	0.001
Coronary artery disease	299 (54%)	141 (53%)	158 (54%)	1.000
Atrial fibrillation	210 (38%)	130 (49%)	80 (27%)	< 0.001
Arterial hypertension	380 (68%)	191 (72%)	189 (64%)	0.046
Diabetes mellitus Type 2	202 (36%)	61 (23%)	141 (48%)	< 0.001
Pulmonary disease	110 (20%)	57 (22%)	53 (18%)	0.338
Serum creatinine, mg/dl	1.1 (0.9–1.3)	1.2 (0. 9–1.4)	1.0 (0.9–1.2)	< 0.001
NT‐proBNP, pg/mL	1256 (613–3512)	1072 (479–3116)	1869 (847–4384)	< 0.001
*NYHA classification*				< 0.001
II	327 (59%)	180 (68%)	147 (50%)	
III	173 (31%)	65 (25%)	108 (37%)	
IV	38 (7%)	9 (3%)	29 (10%)	
*Cardiovascular premedication*				
RAASi	522 (94%)	243 (92%)	279 (95%)	0.227
Beta blocker	519 (93%)	237 (90%)	282 (96%)	0.005
Diuretics	505 (91%)	226 (86%)	279 (95%)	< 0.001
Aldosterone antagonist	432 (77%)	188 (71%)	244 (83%)	0.001
*Echocardiographic parameters*				
LV‐EF, %	36 (30–46)	38 (32–47)	35 (29–45)	0.002
≤40%	373 (67%)	163 (62%)	210 (71%)	
41 bis 49%	102 (18%)	56 (21%)	46 (16%)	
≥50%	83 (15%)	45 (17%)	38 (13%)	
Peak E‐wave velocity, cm/s	90 (62–107)	76 (59–103)	94 (71–108)	< 0.001
E/e' ratio	16 (10 –20)	15 (10–19)	17 (12–21)	0.001
E/A ratio	1.0 (0.7–1.9)	1.0 (0.7–1.7)	1.1 (0.8–2.0)	0.098
DT, ms	0.2 (0.1–0.2)	0.2 (0.2–0.3)	0.2 (0.1–0.2)	< 0.001
LAVI, mL/m²	29 (22–43)	33 (24–43)	26 (20–37)	< 0.001
LV‐EDVI, mL/m²	68 (50–90)	68 (54–92)	70 (46–87)	0.125
LV‐ESVI, mL/m²	44 (28–64)	41 (30–66)	46 (26–63)	0.740
LV‐MI, g/m²	90 (75–123)	90 (76–127)	89 (62–120)	0.240
RWT	0.3 (0.3–0.4)	0.3 (0.3–0.4)	0.3 (0.3–0.4)	0.080
LV‐SI	0.6 (0.5–0.7)	0.6 (0.5–0.7)	0.6 (0.5–0.7)	0.270

Abbreviations: BMI, body mass index; LAVI, left atrial volume index; LV‐EDVI, left ventricular end‐diastolic volume index; LV‐ESVI, left ventricular end‐systolic volume index; LV‐MI, left ventricular mass index; LV‐SI, left ventricular sphericity index; LV‐EF, left ventricular ejection fraction; n., number; NT‐proBNP, N‐terminal pro‐B‐type natriuretic peptide; NYHA, New York Heart Association functional classification; RAASi, Renin‐angiotensin‐aldosterone system inhibitor; RWT, left ventricular relative wall thickness.

### 3.2 Peak E‐Wave Velocity, E/e' Ratio, and E/A Ratio

In the overall study population, there was a significant reduction in peak E‐wave velocity after 1 year (mean difference = −5.88 cm/s, 95% CI: −8.71 to −3.06, *p* < 0.001). This reduction was predominantly driven by the empagliflozin group (mean difference = −12.76 cm/s, 95% CI: −16.26 to −9.27, *p* < 0.001), while the dapagliflozin group showed non‐significant changes (mean difference = 1.74 cm/s, 95% CI: −2.63 to 6.11, *p* = 0.433) (Figure 1).

Regarding the E/e' ratio, the overall population also exhibited a significant decrease after 1 year (mean difference = −1.62, 95% CI: −2.49 to −0.76, *p* < 0.001). This decrease was primarily observed in the empagliflozin group (mean difference = −3.04, 95% CI: −4.17 to −1.91, *p* < 0.001), whereas the dapagliflozin group showed no significant change in the E/e' ratio (mean difference = −0.02, 95% CI: −1.32 to 1.29, *p* = 0.982) (Figure 1).

**Figure 2:**
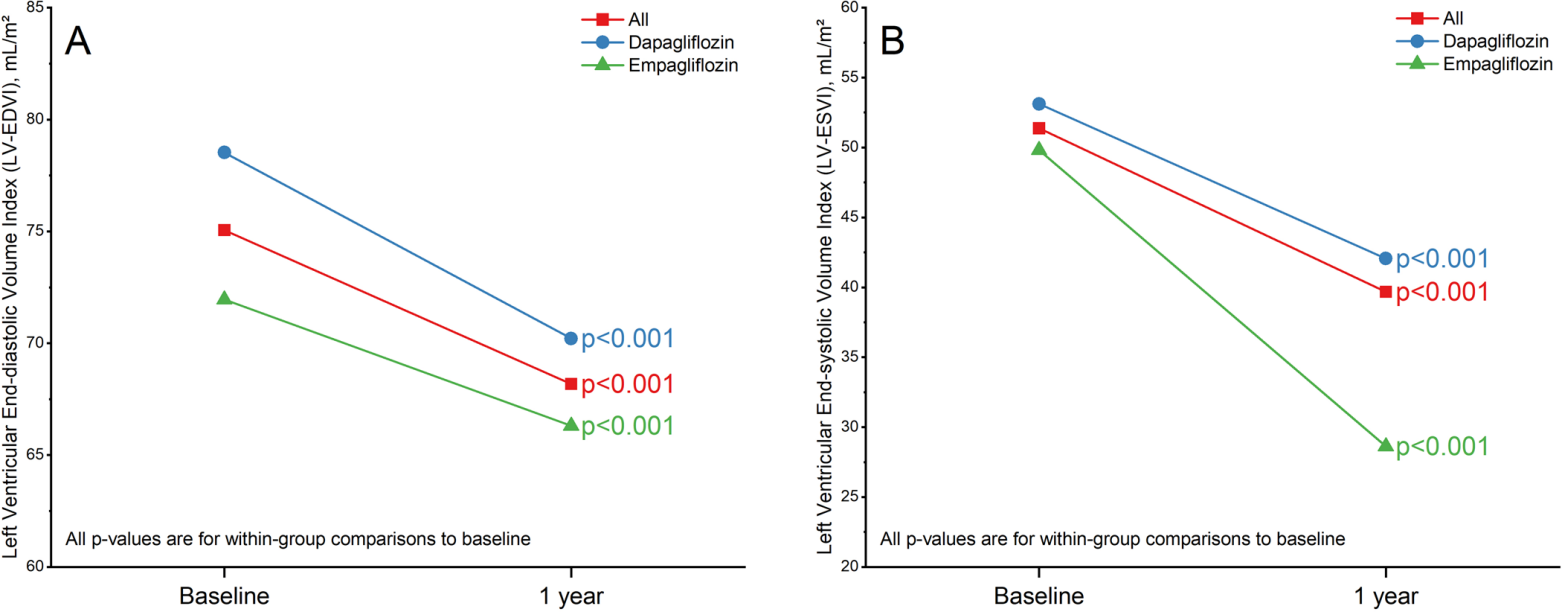
Left ventricular end‐diastolic volume index (LV‐EDVI) (A) and end‐systolic volume index (LV‐ESVI) (B) at baseline and after 1 year. After one year, both dapagliflozin (blue) and empagliflozin (green) groups showed significant reductions in LV‐EDVI and LV‐ESVI (*p* < 0.001 for each group).

Additionally, there was a significant reduction in the E/A ratio across the overall population (mean difference = −0.27, 95% CI: −0.37 to −0.16, *p* < 0.001). In subgroup analysis, both the dapagliflozin group (mean difference = −0.27, 95% CI: −0.38 to −0.15, *p* < 0.001) and the empagliflozin group (mean difference = −0.27, 95% CI: −0.43 to −0.10, *p* = 0.002) demonstrated significant reductions in the E/A ratio over 1 year (Supporting Information S1: Figure 2).

### 3.3 E‐Wave Deceleration Time

A significant increase in E‐wave deceleration time was noted across participants after 1 year (mean difference = 43 ms, 95% CI: 34 to 52, *p* < 0.001). This increase was observed in both the dapagliflozin group (mean difference = 32 ms, 95% CI: 20 to 44, *p* < 0.001) and the empagliflozin group (mean difference = 52 ms, 95% CI: 39 to 65, *p* < 0.001, Supporting Information S1: Figure 2).

### 3.4 LAVI

**Figure 3:**
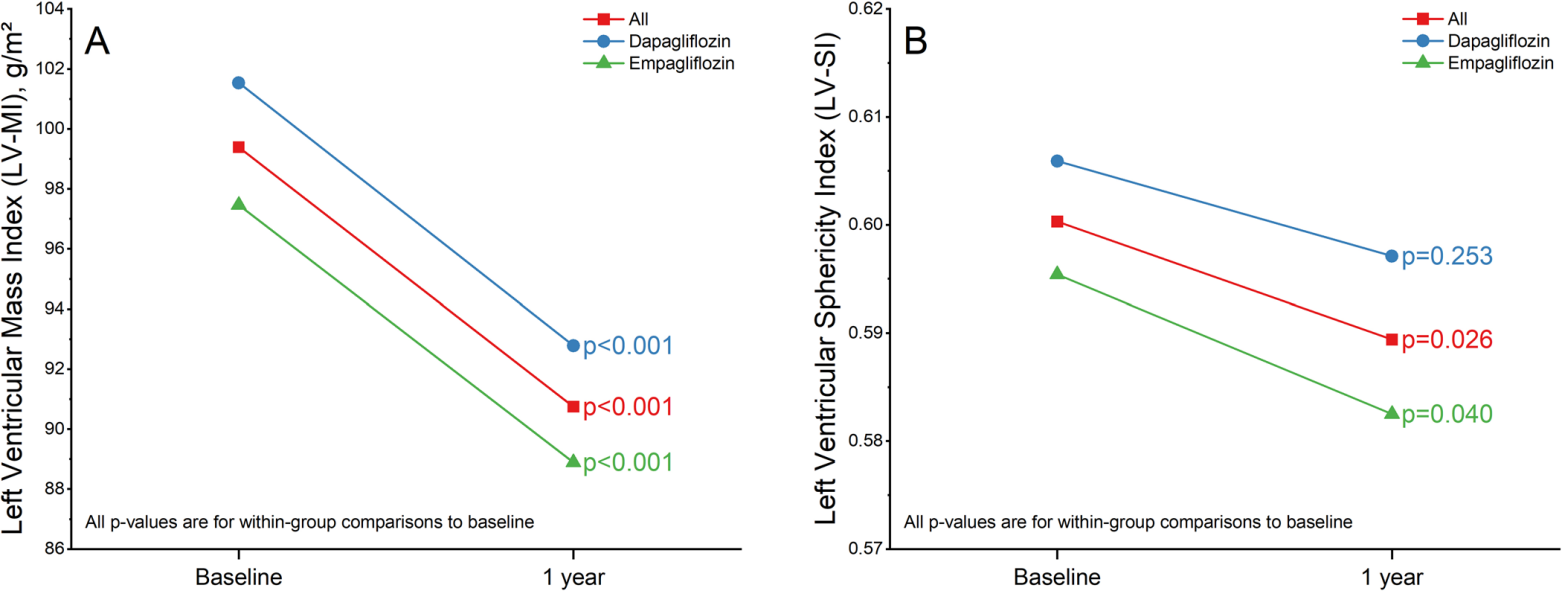
Left ventricular mass index (LV‐MI) (A) and left ventricular sphericity index (LV‐SI) (B) at baseline and after one year. After one year, significant reductions in LV‐MI were observed in both the dapagliflozin (blue) and empagliflozin (green) groups. Significant reductions in LV‐SI were observed only in the empagliflozin group (*p* = 0.040), with no statistically significant change in the dapagliflozin group (*p* = 0.253).

A significant reduction in LAVI was observed overall after 1 year (mean difference = −3.51 mL/m², 95% CI: −4.70 to −2.32, *p* < 0.001). This reduction was seen in both the dapagliflozin group (mean difference = −4.27 mL/m², 95% CI: −6.01 to −2.53, *p* < 0.001) and the empagliflozin group (mean difference = −2.80 mL/m², 95% CI: −4.43 to −1.17, *p* = 0.001) (Supporting Information S1: Figure 3).

### 3.5 LV Volume Indices

In the overall study population, there was a significant reduction in the LV‐EDVI and LV‐ESVI after 1 year. The LV‐EDVI decreased by an average of −6.55 mL/m² (95% CI: −8.58 to −4.51, *p* < 0.001). This reduction was observed in both the dapagliflozin group, with a mean difference of −8.32 mL/m² (95% CI: −11.14 to −5.50, *p* < 0.001), and the empagliflozin group, which showed a mean difference of −4.92 mL/m² (95% CI: −7.84 to −2.00, *p* = 0.001) (Figure 2).

Similarly, the LV‐ESVI also showed a significant decrease, with a mean difference of −11.33 mL/m² (95% CI: −13.23 to −9.44, *p* < 0.001). The reduction was consistent in both the dapagliflozin group (mean difference = −11.06 mL/m², 95% CI: −13.92 to −8.20, *p* < 0.001) and the empagliflozin group (mean difference = −11.58 mL/m², 95% CI: −14.11 to −9.05, *p* < 0.001) (Figure 2).

### 3.6 LV‐MI

Significant reduction in LV‐MI was observed across the study population after 1 year (mean difference = −8.75 g/m², 95% CI: −11.43 to −6.07, *p* < 0.001). This reduction was consistent in both the dapagliflozin group (mean difference = −8.96 g/m², 95% CI: −13.20 to −4.72, *p* < 0.001) and the empagliflozin group (mean difference = −8.56 g/m², 95% CI: −11.95 to −5.17, *p* < 0.001) (Figure 3).

### 3.7 LV RWT

Throughout the study period, there was no significant change in RWT after 1 year (mean difference = 0.013, 95% CI: −0.001 to 0.027, *p* = 0.063). Neither the dapagliflozin group (mean difference = 0.009, 95% CI: −0.005 to 0.024, *p* = 0.214) nor the empagliflozin group (mean difference = 0.016, 95% CI: −0.006 to 0.039, *p* = 0.155) showed significant alterations in RWT (Supporting Information S1: Figure 4).

### 3.8 LV‐SI

In the overall study population, there was significant reduction in LV‐SI after 1 year (mean difference = −0.01, 95% CI: −0.02 to −0.001, *p* = 0.026). In subgroup analyses, the dapagliflozin group showed a non‐significant reduction (mean difference = −0.01, 95% CI: −0.02 to 0.006, *p* = 0.253), whereas the empagliflozin group demonstrated a significant reduction (mean difference = −0.01, 95% CI: −0.02 to −0.0005, *p* = 0.040) (Figure 3).

### 3.9 LV‐EF and NT‐proBNP

There was a significant increase in LVEF overall after 1 year (mean difference = 7.15%, 95% CI: 6.29 to 8.02, *p* < 0.001). In subgroup analyses, the dapagliflozin group showed a significant improvement in LVEF (mean difference = 5.14%, 95% CI: 4.06 to 6.22, *p* < 0.001), while the empagliflozin group demonstrated an even greater increase (mean difference = 9.00%, 95% CI: 7.70 to 10.29, *p* < 0.001). Additionally, there was a significant reduction in NT‐proBNP levels across both groups, with a significant decrease observed in the dapagliflozin group (*Z* = −10.80, *p* < 0.001) and the empagliflozin group (*Z* = −11.42, *p* < 0.001) (Supporting Information S1: Figure 3).

### 3.10 Mortality and HF‐Related Hospitalization

During the median follow‐up period of 12 months (IQR: 10–17 months), 106 patients (19%) were hospitalized at least once for HF. This included 57 patients (22%) in the dapagliflozin group and 49 patients (17%) in the empagliflozin group. There was no significant difference in the time to first HF‐related hospitalization between the dapagliflozin and empagliflozin groups (HR: 1.30, 95% CI: 0.87 to 1.93, *p* = 0.201). A total of 14 patients (3%) experienced all‐cause mortality, with six patients (2%) in the dapagliflozin group and eight patients (3%) in the empagliflozin group. There was no significant difference in the time to all‐cause mortality between the two groups (HR: 0.63, 95% CI: 0.21 to 1.93, *p* = 0.416). Additionally, no significant difference was observed in the time to the combined endpoint of all‐cause mortality and first HF‐related hospitalization between dapagliflozin and empagliflozin (HR: 1.14, 95% CI: 0.82 to 1.59, *p* = 0.425).

## 4 Discussion

Over the 1‐year follow‐up, significant improvements were observed in key echocardiographic measures, including reductions in the E/A ratio, E‐wave deceleration time, LAVI, LV end‐diastolic and end‐systolic volumes (LV‐EDVI, LV‐ESVI), LV‐MI, and ejection fraction (LVEF). Reductions in peak E‐wave velocity, E/e' ratio, and LV‐SI were significant only in the empagliflozin group. No significant changes in RWT were observed with either dapagliflozin or empagliflozin. Furthermore, no differences were found between the two groups regarding HF hospitalizations or all‐cause mortality.

Data on the effects of SGLT2 inhibitors (SGLT2is) on RWT are limited. Most studies examining SGLT2is' impact on myocardial structure focus on LV mass [9]. Since LV mass calculations include LV cavity diameter, reductions in LV mass may largely reflect decreases in cavity size rather than wall thickness. Nevertheless a subanalysis of the EMPA‐TROPISM study did report significant reductions in both extracellular and cardiomyocyte volumes with empagliflozin, as assessed by cardiac magnetic resonance imaging, suggesting potential reductions in LV hypertrophy over time [14]. However, EMPA‐TROPISM was not primarily designed to evaluate the impact of SGLT2is on wall thickness. In addition this subanalysis lacked baseline data on septal, posterior, or RWT parameters, as well as the degree of LV hypertrophy in the examined study population [6]. Furthermore, the EMPA‐TROPISM subanalysis was limited by a small sample size of 29 patients and was conducted at a single site, which restricts the generalizability of its findings [6]. By contrast, our study involved a considerably larger cohort of 558 patients and a longer follow‐up period of 1 year, providing a more comprehensive assessment of SGLT2is' effects on LV remodeling parameters, including a detailed evaluation of LV wall thickness.

In the current study, significant reductions in LV remodeling parameters, including LV volumes, mass, sphericity indexes, and ejection fraction, were observed with SGLT2is. However, previous studies have reported mixed results regarding the effects of SGLT2is on LV remodeling. For example, a meta‐analysis by Theofilis et al. reported a neutral effect on LV end‐diastolic volume (LVEDV) despite a reduction in LV end‐systolic volume (LVESV) with SGLT2is [9]. In contrast, randomized controlled trials focusing on HF patients, such as SUGAR‐DM‐HF, EMPA‐TROPISM, and Empire HF, have demonstrated reductions in LV volume indexes with SGLT2is, aligning with our findings [6, 15, 16]. This discrepancy may be attributed to differences in patient populations, as most studies in the Theofilis et al. meta‐analysis involved patients without HF [9]. Such variations highlight the importance of distinguishing between studies that examine the mechanisms of action of SGLT2is in patients with and without HF.

Improvements in LV remodeling are widely recognized as a key mechanism through which pharmacological therapies enhance clinical outcomes in HF patients. Previous studies have suggested that even a modest 5% reduction in LV volumes may correspond to as much as a 20% reduction in the combined endpoint of death or hospitalization for HF [17]. While the exact mechanisms behind these LV remodeling benefits with SGLT2is remain uncertain, several hypotheses offer possible explanations. One proposed mechanism involves the diuretic‐like effect of SGLT2is [18, 19]. By promoting increased urinary excretion of sodium and glucose through inhibition of their reabsorption in the proximal renal tubules, SGLT2is reduce plasma volume, thereby lowering cardiac preload. The observed decreases in peak E velocity and prolongation of deceleration time in our study support this preload‐reducing effect. In line with our findings, studies such as EMPA‐RESPONSE‐AHF and RECEDE‐CHF have demonstrated increased urine output and reduced diuretic requirements in HF patients treated with empagliflozin [18, 19]. Additionally, the DAPA‐HF trial reported that HF patients on dapagliflozin were more likely to experience reduced diuretic doses and less likely to require diuretic escalation over an 18‐month period [3].

However, previous research by Yasui et al. and Heise et al. suggests that the diuretic effect of SGLT2is may be transient, challenging the preload reduction theory. These discrepancies could stem from differences in patient populations, as both studies involved patients with type 2 diabetes without HF [20, 21]. In non‐HF patients, compensatory mechanisms may limit the diuretic response, while in HF patients, co‐prescription with loop diuretics might enhance the overall effect of SGLT2is [19].

Another hypothesis suggests that SGLT2is promote a metabolic shift from glucose to ketone bodies, which serve as a more efficient energy source, thereby enhancing myocardial efficiency [22]. While the exact mechanisms driving these favorable effects on cardiac remodeling are uncertain, the reduction in NT‐proBNP observed with SGLT2is in our study aligns with this proposed action. NT‐proBNP is a surrogate marker for the degree of LV wall stress, and its reduction is associated with improved health status and outcomes in HF patients [23, 24, 25].

However, the impact of SGLT2is on NT‐proBNP levels is also controversial. In the Empire HF trial, for instance, empagliflozin led to reduced LV volumes but did not significantly affect NT‐proBNP levels [16]. Nevertheless, this lack of significant change could be due to the limited sample size of 95 patients and the relatively short follow‐up period of only 12 weeks [16]. Similarly, the DEFINE‐HF trial, which assessed dapagliflozin in patients with HFrEF, did not demonstrate significant reductions in adjusted mean NT‐proBNP levels over 12 weeks compared to placebo [26]. A suggested explanation for this finding was that the established HFrEF and high baseline NT‐proBNP levels in the examined cohort may have precluded substantial changes in NT‐proBNP levels [26]. These findings underscore the complexity of responses to SGLT2is and highlight the need for further research to elucidate how these drugs influence clinical outcomes in HF.

The significant reductions in peak E‐wave velocity, E/e' ratio, and LV‐SI observed with empagliflozin, but not with dapagliflozin, in our study may reflect differences in efficacy or mechanisms of action between these medications. The meta‐analysis by Theofilis et al. also found that dapagliflozin was associated with smaller reductions in LVEDV and LVESV and no significant changes in LV mass index (LVMI) when compared to empagliflozin [9]. However, as previously mentioned, this meta‐analysis primarily included studies examining patients without HF [9]. Similarly, in the REFORM trial, no significant change in LVEDV or LVEDV was also observed with dapagliflozin over 1 year in HF patients [8]. This lack of effect could potentially be attributed to the limited sample size of only 28 patients. Additionally, all participants in the REFORM trial had diabetes, and the cohort had a higher mean LVEF of 46% ± 12% and a higher mean body mass index (BMI) of 32.5 kg/m² compared to our study cohort [8]. These findings underscore the potential variability among different SGLT2is.

In a large multicenter retrospective study involving 28,075 HF patients, those treated with empagliflozin were less likely to experience the composite outcome of all‐cause mortality or hospitalization compared to patients on dapagliflozin [27]. Similarly, a meta‐analysis of 16 randomized controlled trials reported significant reductions in systolic blood pressure with empagliflozin but not with dapagliflozin in HF patients, further supporting the possibility of differing efficacy or effect between these drugs [28]. To clarify these differences and optimize therapeutic strategies in HF management, comparative randomized controlled trials directly assessing the effects of different SGLT2is are essential.

## 5 Limitations

This study, while providing valuable insights, has certain limitations. As a retrospective analysis, it is prone to selection bias and potential confounding. The lack of randomization limits our ability to establish a definitive association between SGLT2is and the observed echocardiographic changes. Significant differences in baseline characteristics between the groups also made propensity score matching challenging to achieve. Our follow‐up period was limited to 1 year, offering robust short‐term data but possibly missing the longer‐term effects of empagliflozin and dapagliflozin on cardiac structure and function. Additionally, while echocardiographic parameters are widely accepted markers of cardiac remodeling, they may not capture the full spectrum of changes associated with SGLT2i therapy and are subject to investigator bias. Nonetheless, the significant findings in this large cohort underscore the need for extended follow‐up and larger, randomized comparative trials, ideally incorporating cardiac magnetic resonance, to further explore the differential effects of empagliflozin and dapagliflozin on cardiac structure and function.

## 6 Conclusion

Empagliflozin demonstrated more pronounced effects on LV remodeling markers, including peak E‐wave velocity, E/e' ratio, and LV‐SI, compared to dapagliflozin. These findings suggest potential efficacy differences between SGLT2is, highlighting the need for future randomized comparative studies.

## Author Contributions

M.B. and M.U.B. conceptualized and designed the study. M.B. and M.H. collected and interpreted patient data. M.B. wrote the first draft of the manuscript. M.B., M.H., and M.R. performed the statistical analysis. K.I. and A.Y. provided supervision and reviewed and edited the manuscript. All authors had full access to all the data in the study, reviewed and edited the manuscript, approved the final version of the manuscript, and had final responsibility for the decision to submit for publication.

## Consent

The authors have nothing to report.

## Conflicts of Interest

The authors declare no conflicts of interest.

## Supporting information


**Supplemental Figure 1:** Flow Chart. **Supplemental Figure 2:** E‐wave deceleration time (A) and E/A ratio (B) at baseline and after one year. **Supplemental Figure 3:** Left Atrial Volume Index (LAVI) (A), Left Ventricular Ejection Fraction (LVEF) (B), and NT‐proBNP levels (C) at baseline and after one year. **Supplemental Figure 4:** Left Ventricular Relative Wall Thickness (RWT) at baseline and after one year.

## Data Availability

The data that support the findings of this study are available on request from the corresponding author. The data are not publicly available due to privacy or ethical restrictions.
