# Supplementary material for: Impact of Empagliflozin Versus Dapagliflozin on Left Ventricular Remodeling in Heart Failure Patients: A 1‐Year Comparative Study
Source: Clin Cardiol. 2025 Sep 18;48(9):e70192. doi: 10.1002/clc.70192 (PMC12445616; doi:10.1002/clc.70192)

**Supplemental Material**

**Supplemental Figure 1: Flow Chart.**

**Supplemental Figure 2: E-wave deceleration time (A) and E/A ratio (B) at baseline and after one year.**

After one year, significant increases in E-wave deceleration time were observed across both the dapagliflozin (blue, p<0.001) and empagliflozin (green, p<0.001) groups. Similarly, reductions in E/A ratio were significant for both dapagliflozin (p=0.001) and empagliflozin (p=0.002).


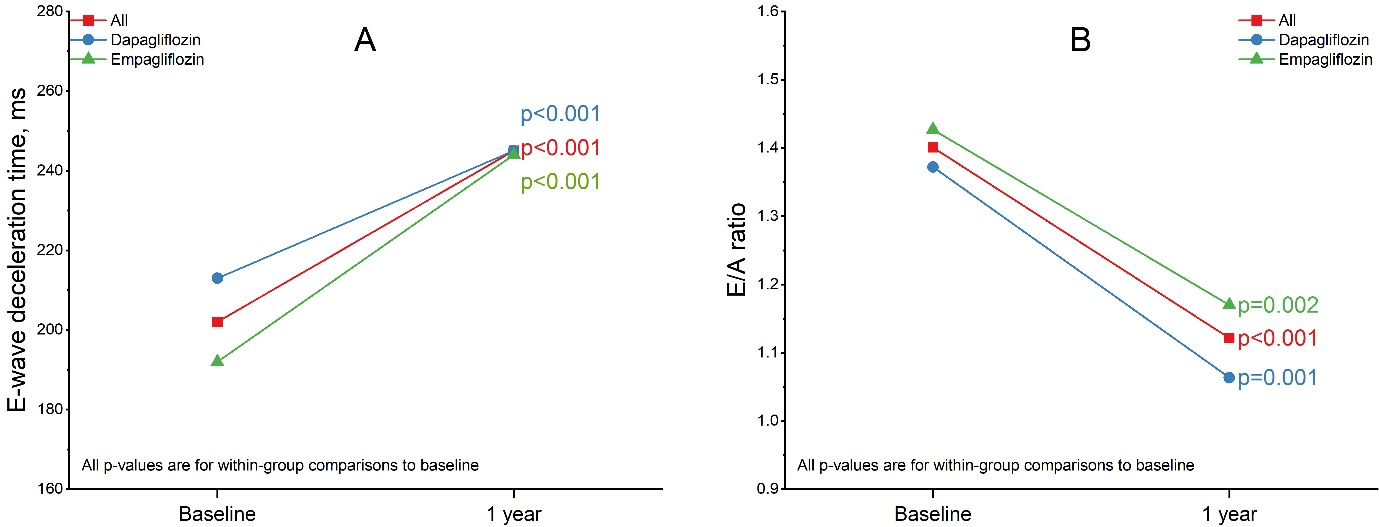


**Supplemental Figure 3: Left Atrial Volume Index (LAVI) (A), Left Ventricular Ejection Fraction (LVEF) (B), and NT-proBNP levels (C) at baseline and after one year.**

After one year, significant reductions in LAVI, LVEF and NT-proBNP levels were observed in both dapagliflozin (blue) and empagliflozin (green) groups.


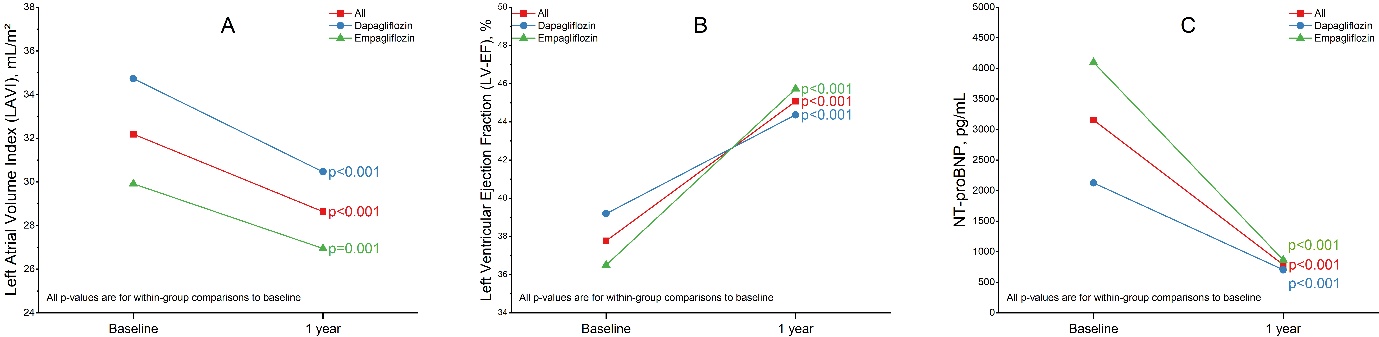


**Supplemental Figure 4: Left Ventricular Relative Wall Thickness (RWT) at baseline and after one year.**

No significant changes in RWT were observed after one year in either group: dapagliflozin (blue, p=0.214) or empagliflozin (green, p=0.155).


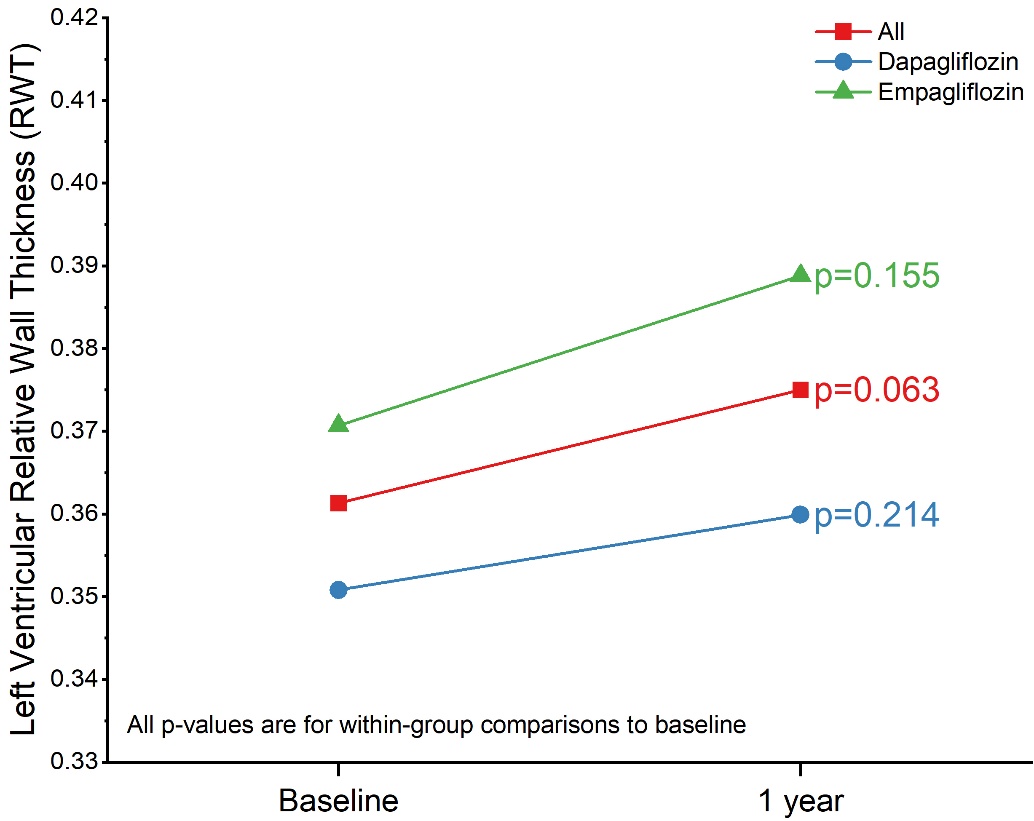

Supplement: Supplementary file 1 — Supplemental Figure 1: Flow Chart. Supplemental Figure 2: E‐wave deceleration time (A) and E/A ratio (B) at baseline and after one year. Supplemental Figure 3: Left Atrial Volume Index (LAVI) (A), Left Ventricular Ejection Fraction (LVEF) (B), and NT‐proBNP levels (C) at baseline and after one year. Supplemental Figure 4: Left Ventricular Relative Wall Thickness (RWT) at baseline and after one year. [file CLC-48-e70192-s001.docx]
